# Microbiome-derived antimicrobial peptides show therapeutic activity against the critically important priority pathogen, *Acinetobacter baumannii*

**DOI:** 10.1038/s41522-024-00560-2

**Published:** 2024-09-30

**Authors:** P. J. Alexander, L. B. Oyama, H. Olleik, F. Godoy Santos, S. O’Brien, A. Cookson, S. A. Cochrane, B. F. Gilmore, M. Maresca, S. A. Huws

**Affiliations:** 1 Institute for Global Food Security, School of Biological Sciences, Queen’s University Belfast, Belfast, UK; 2 Aix Marseille Univ, CNRS, Centrale Marseille, iSm2 (UMR7313), Marseille, France; 3 School of Pharmacy, QUB, Medical Biology Centre, Belfast, UK; 4 Institute of Biological, Environmental and Rural Sciences, Aberystwyth University, Aberystwyth, UK; 5 School of Chemistry and Chemical Engineering, Queen’s University Belfast, Belfast, UK

**Keywords:** Pathogens, Antimicrobials

## Abstract

*Acinetobacter baumannii* is designated by the World Health Organisation as a critical priority pathogen. Previously we discovered antimicrobial peptides (AMPs), namely Lynronne-1, -2 and -3, with efficacy against bacterial pathogens, such as *Staphylococcus aureus* and *Pseudomonas aeruginosa*. Here we assessed Lynronne-1, -2 and -3 structure by circular dichroism and efficacy against clinical strains of *A. baumannii*. All Lynronne AMPs demonstrated alpha-helical secondary structures and had antimicrobial activity towards all tested strains of *A. baumannii* (Minimum Inhibitory Concentrations 2–128 μg/ml), whilst also having anti-biofilm activity. Lynronne-2 and -3 demonstrated additive effects with amoxicillin and erythromycin, and synergy with gentamicin. The AMPs demonstrated little toxicity towards mammalian cell lines or *Galleria mellonella*. Fluorescence-based assay data demonstrated that Lynronne-1 and -3 had higher membrane-destabilising action against *A. baumannii* in comparison with Lynronne-2, which was corroborated by transcriptomic analysis. For the first time, we demonstrate the therapeutic activity of Lynronne AMPs against *A. baumannii*.

## Introduction

*Acinetobacter baumannii* is a Gram-negative rod-shaped aerobic bacterium, with an ability to adapt to a diverse range of environments^1^. It is most commonly identified clinically as a cause of bacteraemia, urinary tract infections and ventilator-associated pneumonia^2^. Indeed, it was estimated in 2019 that 2.5% of all hospital-acquired infections within Europe, Eastern Mediterranean and Africa were due to *A. baumannii*, with that percentage rising to >5% in intensive care-linked hospital-acquired infections^3^. Reports of infections linked to complex cases such as bacteriaemia-linked arthritis and transmission in healthy adults have also been observed^4,5^. *A. baumannii* is also known for its propensity to develop and acquire resistance to current antimicrobial agents (notably the aminoglycoside and carbapenems families in recent years), demonstrating individual, multidrug and pandrug resistance in clinical settings^6,7^. Due to its adaptability and prevalence, *A. baumannii* has been named as a bacterial species of critical importance, being highlighted on the World Health Organisation's list of priority pathogens as well as being designated as a ESKAPE pathogen by the Infectious Diseases Society of America^8–10^.

Whilst sensitive strains of *A. baumannii* exist towards carbapenems, aminoglycosides and tetracyclines, the likelihood of such easily treatable infections is low. Treatment of drug-resistant *A. baumannii* infections has often been limited to last resort compounds, such as the polymyxins, notably colistin, or cefiderocol. Colistin is often utilised in conjunction with other antimicrobial compounds (predominantly meropenem or ampicillin-sulbactam) in the treatment of *A. baumannii* infections to prevent heteroresistance and improve patient outcomes^11,12^. Cefiderocol (a siderophore cephalosporin), has been indicated as a possible treatment of carbapenem-resistant *A. baumannii*, with good activity reported in lab conditions^10^. Even with such treatment options available, these often have serious side effects, for example, polymyxins can induce nephrotoxicity, and the recommended high doses of ampicillin-sulbactam and minocycline increase the risks of side effects (notably hepatotoxicity)^13^. Additionally, despite only being approved for use in 2020, clinical isolates of *A. baumannii* have already been reported to demonstrate cefiderocol resistance^14^.

Development of novel treatment options, such as those provided by cationic antimicrobial peptides (AMPs), is necessary to ensure effective treatment of *A. baumannii* infections. AMPs have been identified as an alternative to conventional antibiotics since the identification of nisin in 1947, with many demonstrating activities against a variety of pathogenic organisms^15–18^. More importantly, some have been shown to interfere with biofilm formation, which is a major factor in the development of antimicrobial resistance in bacteria^19,20^. The antimicrobial activity of cationic AMPs is attributed to two common features; 1) an overall positive charge^21^, which allows binding to negatively charged bacterial cell membranes and 2) hydrophobic regions necessary for interacting with the lipid components of bacterial membranes. The rapid activity and low resistance development observed for many AMPs make them ideal candidates for novel therapeutic application^22^.

Numerous AMPs identified from the rumen microbiome, including Lynronne peptides, have shown proven efficacy against a variety of bacterial species, including Gram-positive and negative isolates^23–27^. Lynronne-1, -2, and -3 were first identified in 2017 via functional screening of a rumen metagenomic library, and subsequently demonstrated high potential for treating multi-drug resistant infections^26^. Previous studies suggest that they work as membrane disruptors against *S. aureus*^23,25^ and *Pseudomonas aeruginosa*, and possess low mammalian cell cytotoxicity^25^. Lynronne-1 has also undergone structural investigations, which concluded that it displayed an alpha-helical conformation in the presence of bacterial lipids^28^. In addition, these AMPs have been observed to have antibiofilm activity against methicillin-resistant *S. aureus* at 2× minimum inhibitory concentration (MIC)^26^.

In this study, we investigated the efficacy, safety and mechanisms of action of Lynronne-1, -2 and -3 against clinical strains of *A. baumannii*. Consequently, this study provides the first fundamental pre-clinical data needed to determine the feasibility of using these AMPs as innovative therapeutics to treat *A. baumannii* infections.

## Results

### AMP structural analysis

**Fig. 1:**
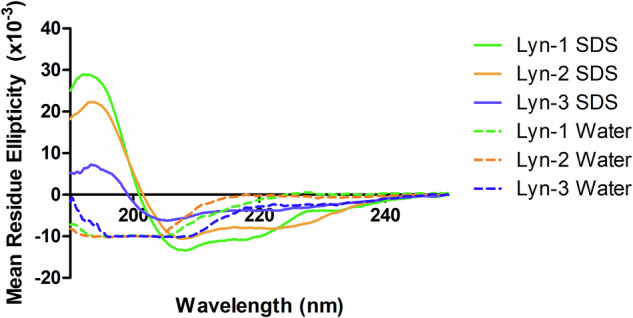
Mean residue ellipticity of the Lynronne antimicrobial peptides in sterile water and 30 mM SDS, collected by far-UV circular dichroism (CD) spectrophotometry. All AMPs were at 20 μg/ml, and the CD spectrum was collected between 185 and 250 nm. Dotted lines indicate results from Lyn-1, -2, -3 in water, and solid lines indicate results from Lyn-1, -2, -3 in 30 mM SDS. All readings were taken 5 times, and the average result taken for processing.

Secondary structure elucidation via circular dichroism (CD), which analyses absorption of circularly polarised UV wavelengths showed that in 30 mM SDS, Lynronne-1, -2 and -3 produce predominantly alpha-helical secondary structures, with peaks observed at around 195 nm, and two slight dips around 208 nm and 220 nm (Fig. 1). Based on the relative peak sizes, Lynronne-1 is predicted to have the highest α-helical content, with Lynronne-2 demonstrating slightly reduced and Lynronne-3 notably lower α-helical content in comparison. In water, they appeared to lack a stable secondary structure, with dips observed around 200 nm, indicating presentation as dissociated linear chains (Fig. 1).

### Minimal inhibitory concentrations (MIC)

**Fig. 2:**
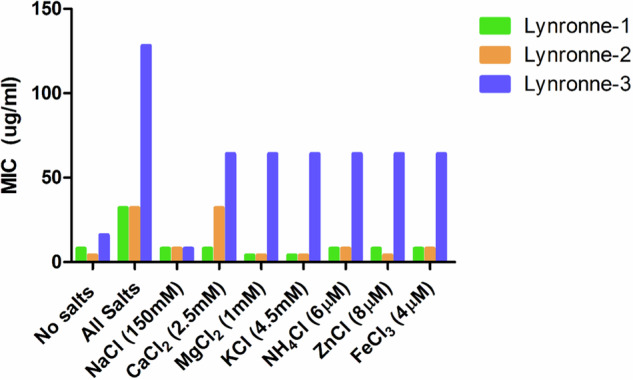
Antimicrobial activity of Lynronne AMPs against *Acinetobacter baumannii* DSM 30007 in presence of physiologic salts. Salts were added into cation-adjusted Mueller Hinton broth, and stated concentrations were identified from previous studies^29^. MICs were performed in triplicate. ‘No salts’ is cation-adjusted MH already containing Ca^2+^ with no additional CaCl_2_ (2.5 mM).

Lynronne-1, -2 and -3 showed inhibitory effects towards all the tested *A. baumannii* strains (Table 1). Lynronne-1 had MIC ranges of 2–16 µg/ml, Lynronne-2; 4–16 µg/ml and Lynronne-3 had a wider range between 8 and 128 µg/ml. Strains DSM 30007, DSM 24110 and S25722 tended to have the lowest MICs and strains DSM 105126 and S27379 the highest tolerance to AMP treatment. Lynronne-3 appeared to have higher MICs against all strains compared with Lynronne-1 and -2 (Table 1). AMPs can lose efficacy in the presence of salts in blood plasma^29^. Therefore, their MICs were further tested in physiological salt conditions. More than 2-fold increases in Lynronne-1 MIC were observed in the presence of calcium chloride (2.5 mM), and 8-fold in Lynronne-2. For Lynronne-3, an 8-fold increase in *A. baumannii* MIC was observed in the presence of most of the tested salts except for 150 mM NaCl, in which no major change was observed (Fig. 2).

**Table 1:** Minimum Inhibitory Concentrations (MICs) of Lynronne-1, -2 and -3 and ciprofloxacin against *Acinetobacter baumannii* strains

Organism Information			Antimicrobial Peptides and comparator antibiotics (µg/ml)			
*Lab no./Strain ID*	*Resistances*	*Lab/Clinical strain*	*Cip*	*L-1*	*L-2*	*L-3*
*DSM 30007/ATCC 19606*	*ND*	*L*	0.5	4	4	8
DSM 30008	ND	L	0.125	4	8	128
DSM 30011	ND	L	0.25	16	8	32
DSM 102929	ND	L	64	2	8	64
DSM 102930	ND	L	32	2	8	64
DSM 105126	ND	L	0.5	16	8	64
DSM 24110	ND	L	0.125	4	4	32
S26063	Sensitive	C	0.5	8	8	64
S15785	OXA-23, OXA-50	C	128	8	8	64
S15908	Sensitive	C	0.25	4	16	64
S27379	IMI, MER	C	0.25	16	16	128
S17658	Sensitive	C	64	16	16	64
S25722	Sensitive	C	0.25	4	4	32
S17910	IMI, MER	C	64	4	8	*32*

Resistances of strains have been listed where identified. MICs were collected in triplicate between 256 and 0.125 μg/ml in cation-adjusted Mueller Hinton broth (MH). The median concentration where no growth was observed after 18–24 h was selected as the MIC.

*Cip* ciprofloxacin, *L1, L2, and L3* Lynronne-1/2/3, *IMI* imipenem, *MER* meropenem, *OXA-23* bla OXA-23 carbapenemase, *OXA-50* bla OXA-50 carbapenemase, *L* laboratory strain, *C* clinical strain, *ND* not determined. Strain IDs beginning with 'S' are clinical strains gifted to us by Public Health Wales, UK.

### Time kill kinetics

**Fig. 3:**
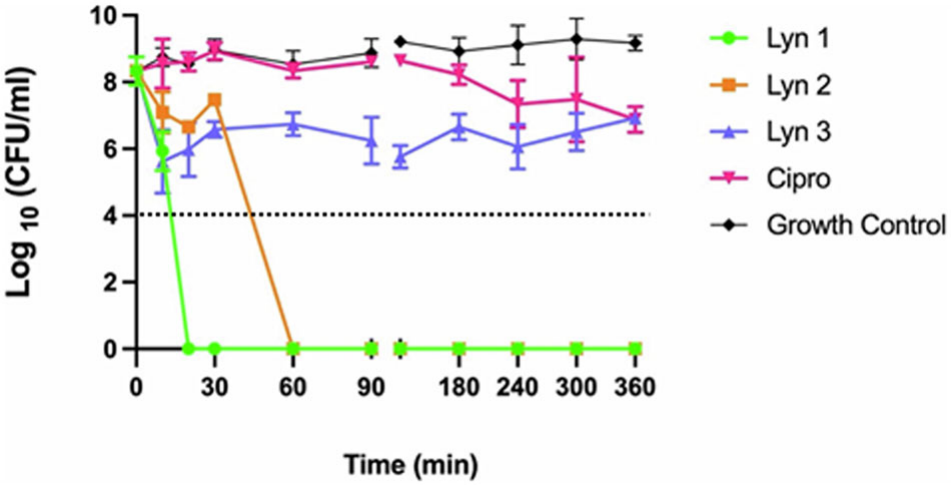
Time-dependent Lynronne AMPs-mediated activity against *Acinetobacter baumannii* DSM 30007. 4× MIC concentrations were used for all antimicrobials, and sterile PBS was used for the growth control. Dotted line indicates detection limit. Plate counts (CFU/ml) were taken at the time intervals of 0, 10, 20, 30, 60 and 1440 min. Broth cultures at a CFU/ml of 10^9^ were incubated at 37 °C, 180 rpm for the duration of the assay. All treatments were tested in triplicate, and plate counts were taken in duplicate. Error bars denote standard deviation from the mean.

We tested the time required for bacteriostatic or bactericidal activities of Lynronne-1, -2 and -3 against the *A. baumannii* type strain DSM 30007. Cell count reductions were seen within 20 min of exposure with >2 log_10_ (CFU/ml) decrease for all AMPs (Fig. 3). These results were in comparison to ciprofloxacin (Cipro), which demonstrated much slower inhibitory activity (as expected, based on the DNA replication targeting mechanism).

### Synergistic assay

The ability of the Lynronne AMPs to produce synergistic effects with conventional antibiotic treatments for *A. baumannii*, as well as vancomycin, which traditionally only targets Gram-positive organisms, was observed through a modified checkerboard assay^30^. Lynronne-1 demonstrated no ability to improve efficacy of any of the tested antimicrobial agents (Table 2) at 0.25x MIC. Lynronne-2 and -3 both demonstrated the same synergistic profile with additive effects with amoxicillin and erythromycin (FIC_a_ of 0.5, 2-fold MIC reduction), and potential synergy with gentamicin (FIC_a_ of 0.25, 4-fold MIC reduction). None of the AMPs showed an ability to induce antimicrobial activity from vancomycin at 0.25× MIC.

**Table 2:** FIC_a_ of selected antimicrobials alongside 0.25 × MIC of the Lynronne peptides

	Lynronne-1	Lynronne-2	Lynronne-3
Amoxicillin	1	0.5	0.5
Erythromycin	1	0.5	0.5
Gentamicin	1	0.25	0.25
Tetracycline	1	1	1
Vancomycin	1	1	1

A checkerboard MIC assay was utilised to determine potential synergistic effects, with antibiotics with varying mechanisms of action. FIC_a_ calculations were used to determine whether there were any indications of additive or synergistic effects, with 1 = no effect, 0.5–0.25 indicating possible additive effects, and <0.25 indicating possible synergy.

### Resistance induction assays

**Fig. 4:**
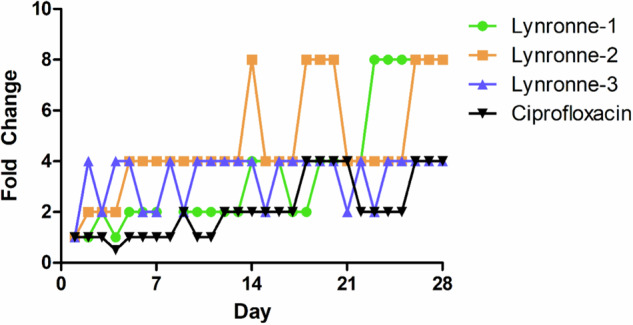
Resistance development during serial passage of *Acinetobacter baumannii* DSM 30007 over 28 days in the presence of sub-inhibitory concentrations of antimicrobials. Wells containing the highest concentration of antimicrobial with growth observed from the most recent overnight MIC test were selected as the starting culture for serial passage. This figure is a representative of the mean change in MIC by 3 biological replicates. Ciprofloxacin was also included as a comparator, and blanks containing sterile MH broth used as a negative control. Fold change is indicative of doubling changes as compared to the initial MIC observed on day 1.

Serial passage in sub-lethal concentrations of each AMP over 28 days showed a slow but steady increase in MIC for Lynronne-1 (4× MIC increase, to 16 µg/ml) and Lynronne-2 (8× increase, to 32 µg/ml), with Lynronne-3 showing a quick 2× increase (to 16 µg/ml) before fluctuating between 8 and 16 µg/ml (Fig. 4). It is unclear at the minute whether these slight increases in MIC are due to a genotypic or phenotypic alteration.

### Activity against biofilms

**Fig. 5:**
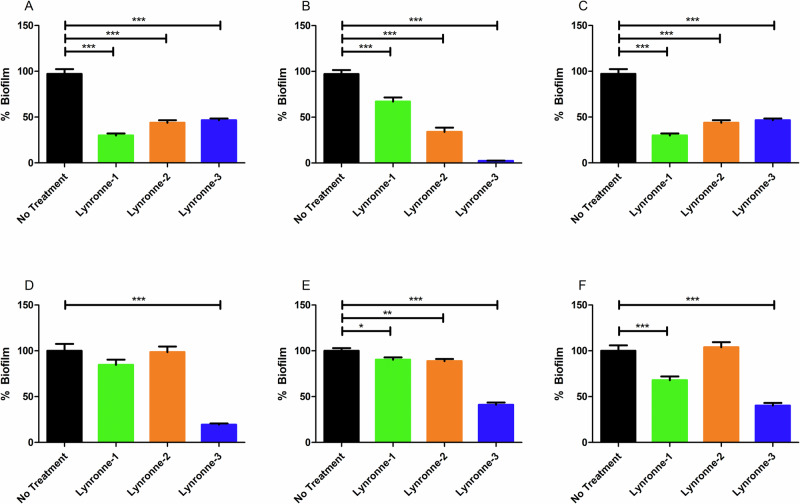
Antibiofilm activity of Lynronne antimicrobial peptides. Effects of the Lynronne antimicrobial peptides on the growth and adhesion of *Acinetobacter baumannii* biofilms (**A**–**C**) and the effects on established biofilms (**D**–**F**). Graphs (**A**, **D**); *A. baumannii* DSM 30007. Graphs (**B**, **E**); *A. baumannii* DSM 102929. Graphs (**C**, **F**); *A. baumannii* clinical S26063. Biofilms were grown in cation-adjusted MH broth for 48 h at 37 °C in 96 well plates. Biofilm mass was determined using crystal violet staining and resolubilisation in acetic acid before OD_600_ readings were taken. Positive controls were established with the inclusion of sterile water, and negative controls were established using sterile cation-adjusted MH broth. Readings were taken with 12 technical replicates, with 3 biological replicates for each assay. Statistically significant differences between treatments and the positive control were determined using 1-way ANOVAs with Dunnett’s post-test. Error bars denote standard deviation.

When examined for their anti-biofilm properties against three strains of *A. baumannii* (chosen due to the fact that they are well studied with genomic data available) with varying abilities to produce biofilm mass (Fig. 5), all 3 AMPs showed varying but significant (*P* < 0.05) abilities to prevent biofilm formation at 4× MIC. Lynronne-1 and Lynronne-2 showed a reduction in biofilm formation of up to 70%. Lynronne 3 showed almost total prevention of biofilm formation by strain DSM 102929, with a reduction of 97%. Lynronne-1, -2 and -3 were also able to disperse established biofilms grown for over 48 h, with Lynronne-1 showing up to 31% reduction against S26063, and Lynronne-2 only demonstrating any significant reduction (11%) against DSM 102929. Lynronne-3 retained the most significant biofilm dispersal/disruption performance, with 60-80% biofilm reduction observed.

### Membrane activity assays, biophysics and transmission electron microscopy

**Fig. 6:**
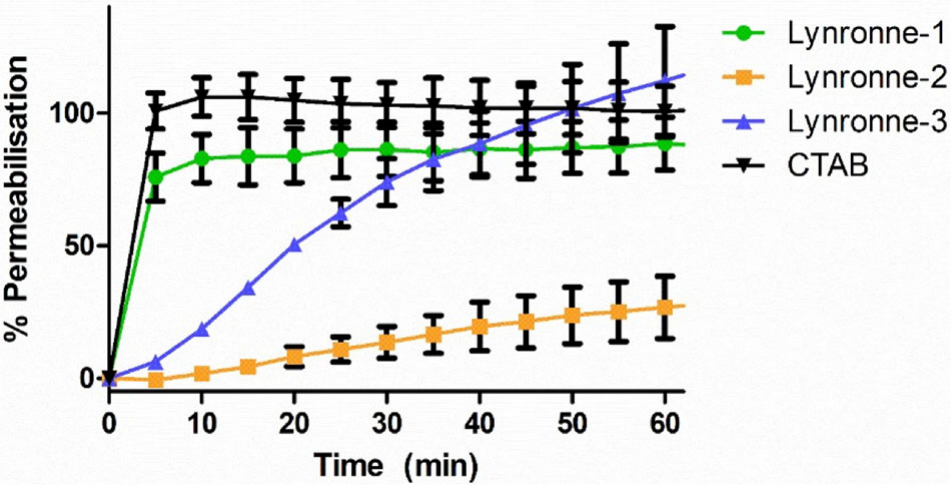
Membrane permeabilisation of Lynronne-1, -2 and -3 over time based on propidium iodide fluorescence at 4× MIC. Increased fluorescence indicates damage/pore formation in the bacterial cell membrane. 100% permeabilisation rate was established using average fluorescence by CTAB control once plateau had been achieved. Readings of excitation/emission at 540 nm and 590 nm were taken in triplicate, and the mean calculated for each time point. Error bars signify SEM as calculated using GraphPad Prism 5. Positive control established by cetrimonium bromide (CTAB) at 300 μM.

**Fig. 7:**
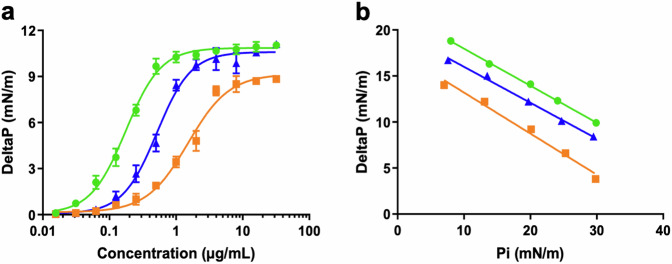
Evaluation of the insertion of the Lynronne AMPs into lipids of *Acinetobacter baumannii.* Green lines: Lynronne-1; orange lines: Lynronne 2; Blue lines; Lynronne 3. The insertion of the Lynronne AMPs into total lipids extract of *A. baumannii* 30007 was measured using the Langmuir film balance (KIBRON apparatus). **a** Evaluation of the dose-dependent insertion of the Lynronne AMPs into monolayer of *A. baumannii* lipids. Lipid monolayers were obtained by spreading lipids extracted from *A. baumannii* 30007 at the water-air interface until reaching an initial surface pressure of 30 ± 0.5 mN/m. Increasing concentrations of Lynronne AMPs were then injected into the water sub-phase and their insertions were measured as the maximal variation of the surface pressure (DeltaP) usually reached within 20–30 min. DeltaP are expressed in mN/m (means ± S.D., *n* = 3). **b** Determination of the critical pressure of insertion. The lipid insertion of the Lynronne AMPs (at 1 µg/mL) was measured using lipid monolayers set-up at different initial surface pressures. Graph was used to calculate the critical pressure of insertion corresponding to the theoretical initial pressure at which no insertion can occurs. The critical pressure of insertion was graphically determined as the intercept of the linear slope with the X-axis when the DeltaP is equal to zero.

**Fig. 8:**
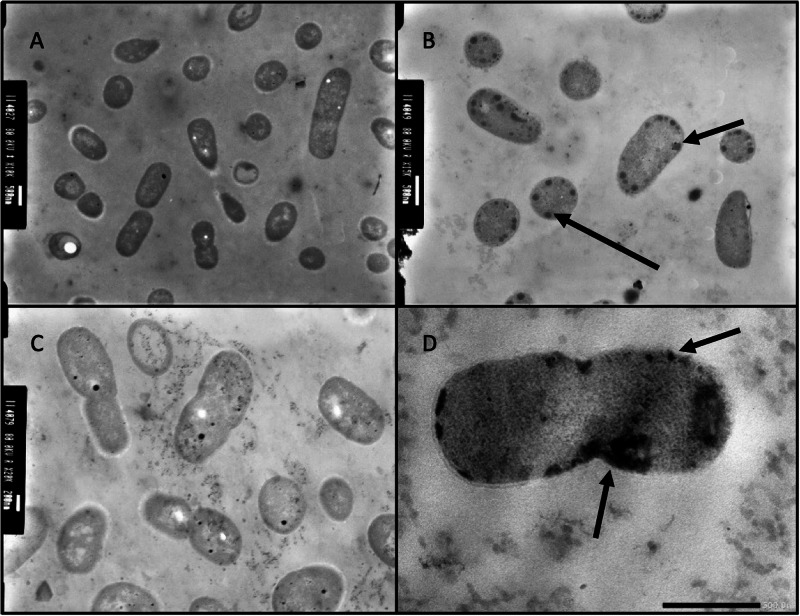
Transmission electron micrographs of *Acinetobacter baumannii* DSM 30007 cells after exposure to Lynronne-1, -2 and -3. **A** untreated cells. **B** Lynronne-1. **C** Lynronne-2. **D** Lynronne-3. All peptides were at 4× MIC concentration, and cells were exposed for 60 min at 37 °C. Scale bars for image (**A**, **B**, **D**) are 500 nm, and image (**C**) is 200 nm. Black arrows in images (**B**, **D**) indicate vacuole aggregation at the bacterial cell membrane, indicating cell damage/response to AMP membrane exposure. These are representative images for each treatment condition.

**Fig. 9:**
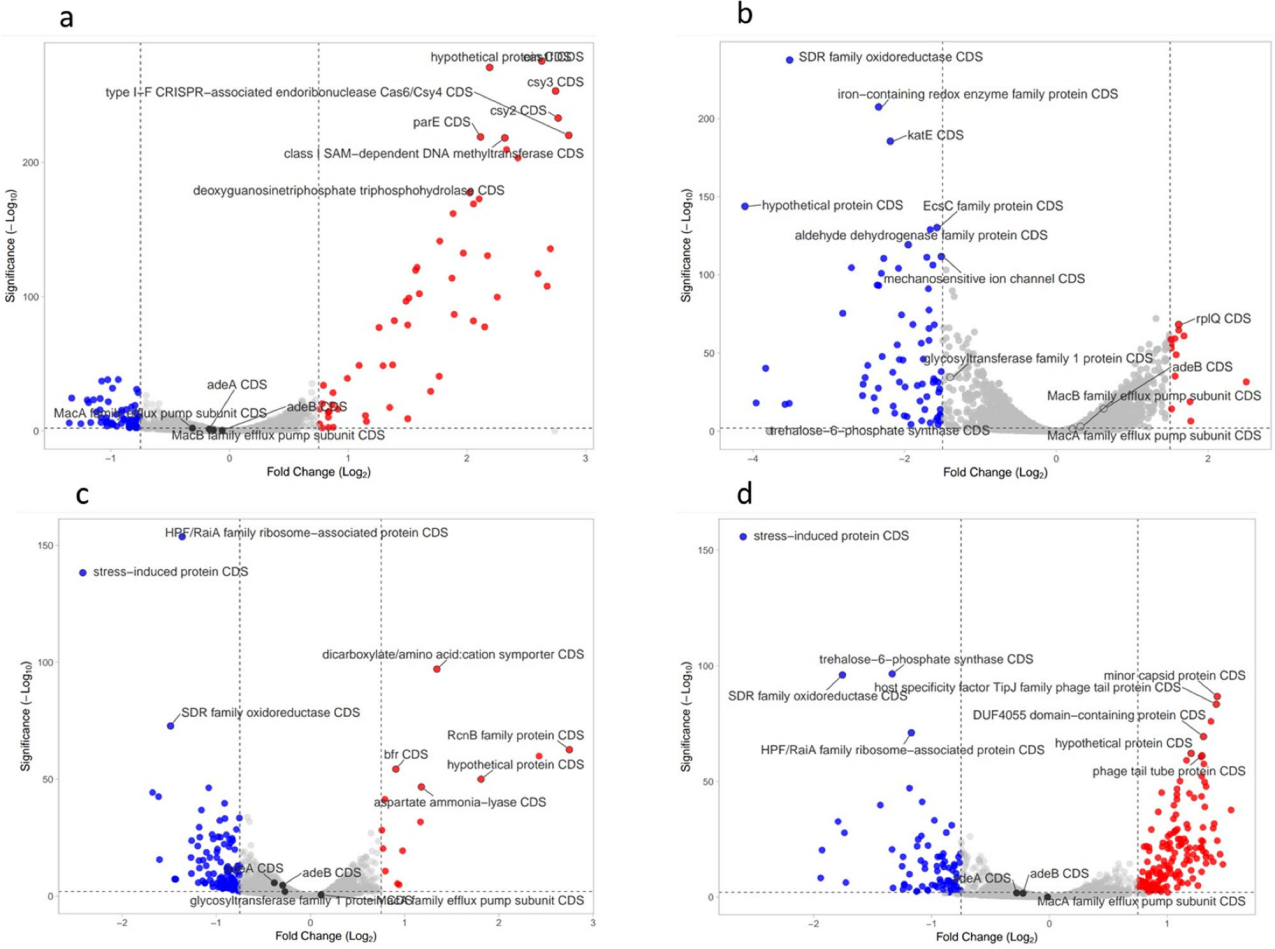
Volcano plot representation of transcriptome change of *A. baumannii* DSM 30,007 cells after exposure to the Lynronne AMPs and ciprofloxacin. **a** Ciprofloxacin. **b** Lynronne-1. **c** Lynronne-2. **d** Lynronne-3. Genes highlighted in blue were significantly downregulated (−log_10_ significance of >2, Log_2_ fold change of >0.75 in either direction). Cell cultures were challenged for 60 min at 1× MIC for all antimicrobials. All treatments were conducted in triplicate, and gene expression counts calculated using Geneious Prime (version 2022.2.2). Gene expression counts were compared for each treatment against the control using DESeq2 using Rstudio, and volcano plots created using VolcaNoseR version 2.0.

The AMPs were tested for their ability to induce membrane permeabilisation, using propidium iodide as a fluorescence-based dye for testing cell membrane integrity^31^. All three tested peptides showed an ability to permeabilise the cell membrane of *A. baumannii* DSM 30007, although Lynronne 2 was noticeably less membrane penetrating in comparison, demonstrating less than 50% permeabilisation within an hour (Fig. 6). This was further explored via lipid insertion biophysics analysis, which indicated that Lynronne-1 and -3 had higher binding affinities (demonstrated by EC_50_ concentrations of 0.175 µg/ml and 0.515 µg/ml respectively, in Table 3) for lipids extracted from *A. baumannii* DSM 30007 as compared to Lynronne-2 (EC_50_ concentrations of 1.512 µg/ml). Biophysics and lipid insertion data corroborate the observation that Lynronne-1 and -3 are more membranolytic than Lynronne-2 (Fig. 7, Table 3). Cell morphology changes due to AMP treatment were observed using transmission electron microscopy (TEM) post exposure at 30, 60 and 120 min. Lynronne-1 and Lynronne-3 displayed numerous vacuole aggregation (indicated by the arrows; Fig. 8B, D) at the cell membrane, but limited morphology changes were observed in the treatment of Lynronne-2 following 60 min exposure (Fig. 9C). At 30 min exposure, cells showed little morphological changes (data not shown). However, at 120 min, there were little or no viable cells for imaging possibly due to cell degradation.

**Table 3:** Biophysics parameters of the insertion of the Lynronne AMPs into lipids from *A. baumannii*

	Lynronne-1	Lynronne-2	Lynronne-3
EC_50_ (µg/mL)	0.175 ± 0.007	1.512 ± 0.106	0.515 ± 0.027
Critical pressure (mN/m)	54.63	39.60	50.94

The parameters of insertion of the Lynronne AMPs into lipids from A. baumannii 30007 were determined from Fig. 8 using GraphPad Prism. The EC50 values (expressed in µg/mL as means ± S.D. (*n* = 3)) were determined from Fig. 8A and correspond to the concentrations of each Lynronne AMP causing 50% of increase in surface pressure compared to the maximal insertion observed for it. The critical pressure values (expressed in mN/m) were determined from Fig. 8B and were graphically determined for each Lynronne AMP as the intercept of the linear slope with the X-axis when the DeltaP is equal to zero.

### Acinetobacter baumannii gene-level response to AMP exposure

The transcriptomic analysis at 1× MIC exposure of *A. baumannii* DSM 30007 to Lynronne-1, -2, -3 and ciprofloxacin highlighted a clear variation in the transcriptome between the different treatments (Fig. 9). 1× MIC exposure for 60 min was chosen to ensure a concentration of AMP was high enough to cause cell inhibition and lysis whilst providing a reasonable timeframe to allow transcriptomic changes in surviving cells to occur. The ciprofloxacin control indicates upregulation in factors linked to DNA replication, with methyltransferases and CRISPR CAS-6/*Csy4* gene upregulation, likely in response to DNA replication inhibition by ciprofloxacin. Lynronne-1 caused an upregulation in the *rpl*- and *rps*-gene families (which are involved in protein synthesis), combined with a number of hypothetical proteins and domains of unknown function (DUFs). Lynronne-2 showed an upregulation in the *RcnB* family protein genes, previously linked to efflux pumps involved in the movement of nickel and copper ions, as well as an increase in a dicarboxylate transporter, indicating links to potential survival mechanisms. Lynronne-3 showed an increase in the same hypothetical proteins and DUFs as Lynronne-1, which were significantly downregulated in ciprofloxacin exposure. Lynronne-2 and -3 also showed a slight upregulation of glycosyltransferase family proteins (involved in cell wall synthesis and modification), and a trehalose-6-phosphate synthase protein, which produces a precursor to trehalose, previously linked to osmotic stress regulation. Conversely, all three peptides showed a marked decrease in a variety of gene families, notably the expression of a stress-induced protein domain, which was highlighted in all three treatments. Other genes of interest downregulated after AMP treatment include the *RaiA* gene family, encoding for hibernation promotion, which was downregulated after exposure to Lynronne-2.

### Toxicity assays

**Fig. 10:**
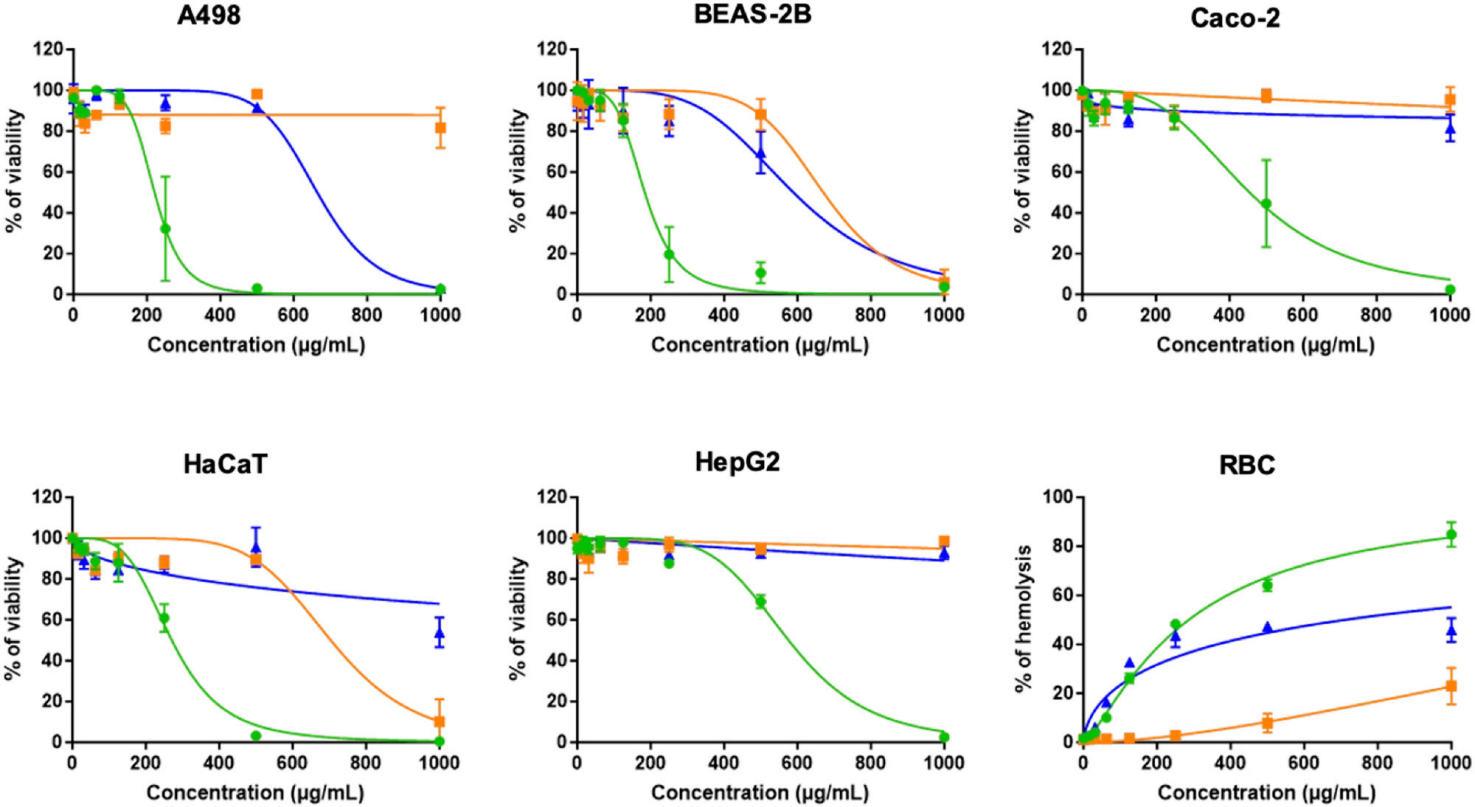
Evaluation of the toxicity of the Lynronne AMPs on human cell lines. The toxicity of the Lynronne AMPs was measured against human cell lines from various organs. Human cells were exposed to increasing concentrations of the Lynronne AMPs. For cytotoxicity determination, A498 (kidney), BEAS-2B (lung), Caco-2 (intestine), HaCaT (skin), and HepG2 (liver) cells, cells were exposed for 48 h before measurement of the cell viability using resazurin assay. Results are expressed as percentage of cell viability using untreated cells as negative controls giving 100% viability. For haemolysis determination, human red blood cells (RBC) were exposed for 1 h before measurement of haemoglobin release. Results are expressed as percentage of haemolysis, using Triton X-100 (at 0.1%) as the positive control giving 100% haemolysis. Results are expressed as means ± S.D. (n3). Green lines: Lynronne-1; orange lines: Lynronne 2; Blue lines; Lynronne 3.

Toxicity of Lynronne AMPs was evaluated using a haemolysis assay with human red blood cells, a resazurin-based cell toxicity assay conducted on human kidney (A498), lung (BEAS-2B), intestinal (Caco-2), liver (HepG2) and skin cells (HaCaT), and the *Galleria mellonella* larval model (Fig. 10). All 3 Lynronne AMPs demonstrated limited toxicity against all of these models. CC_50_ or HC_50_ range from 184.0 to 576.1 µg/mL, from 670.5 to >1000 µg/mL, and from 589.2 to >1000 µg/mL for Lynronne-1, 2, and 3, respectively (Table 4). With MIC values of 2 to 16, 4 to 16, and 8 to 128 µg/mL, this gives therapeutic index (TI) range of 11.5 to288.1, 41.9 to >250, and 4.6 to >125, for Lynronnes 1, 2, and 3, respectively (Table 5). The higher the TI the better the chance that the antimicrobial won’t show toxicity when administered to humans or animals, therefore our AMPs show much promise.

**Table 4:** Toxic effect of the Lynronne AMPs against human cells

	Lynronne-1	Lynronne-2	Lynronne-3
A498 (kidney)	220.7 ± 13.7	>1000	664.1 ± 48.2
BEAS-2B (lung)	184.0 ± 7.9	670.5 ±51.2	589.2 ± 36.4
Caco-2 (intestine)	454.6 + 29.2	>1000	>1000
HaCaT (skin)	271.0 ± 11.1	704.2 ± 47.0	>1000
HepG2 (liver)	576.1 ± 19.4	>1000	>1000
RBC	286.0 ± 8.3	>1000	709.9 ± 122.3

The toxicity of the Lynronne AMPs was evaluated using human cell lines from various organs. The toxic concentrations (either CC_50_ or HC_50_ corresponding to 50% decrease in cell viability or 50% haemolysis, respectively) were determined from Figure Y using GraphPad Prism and are expressed in µg/mL (means ± S.D. (*n* = 3)).

**Table 5:** Therapeutic indexes of the Lynronne AMPs

	Lynronne-1	Lynronne-2	Lynronne-3
MIC range (µg/mL)	2–16	4–16	8–128
Toxicity range (µg/mL)	184.0–576.1	670.5–>1000	589.2–>1000
TI range	11.5–288.1	41.9–>250	4.6–>125

The therapeutic indexes (TI) of Lynronnes were calculated by dividing their range of CC_50_ or HC_50_ obtained on human cells by their range of MIC values on bacteria.

**Fig. 11:**
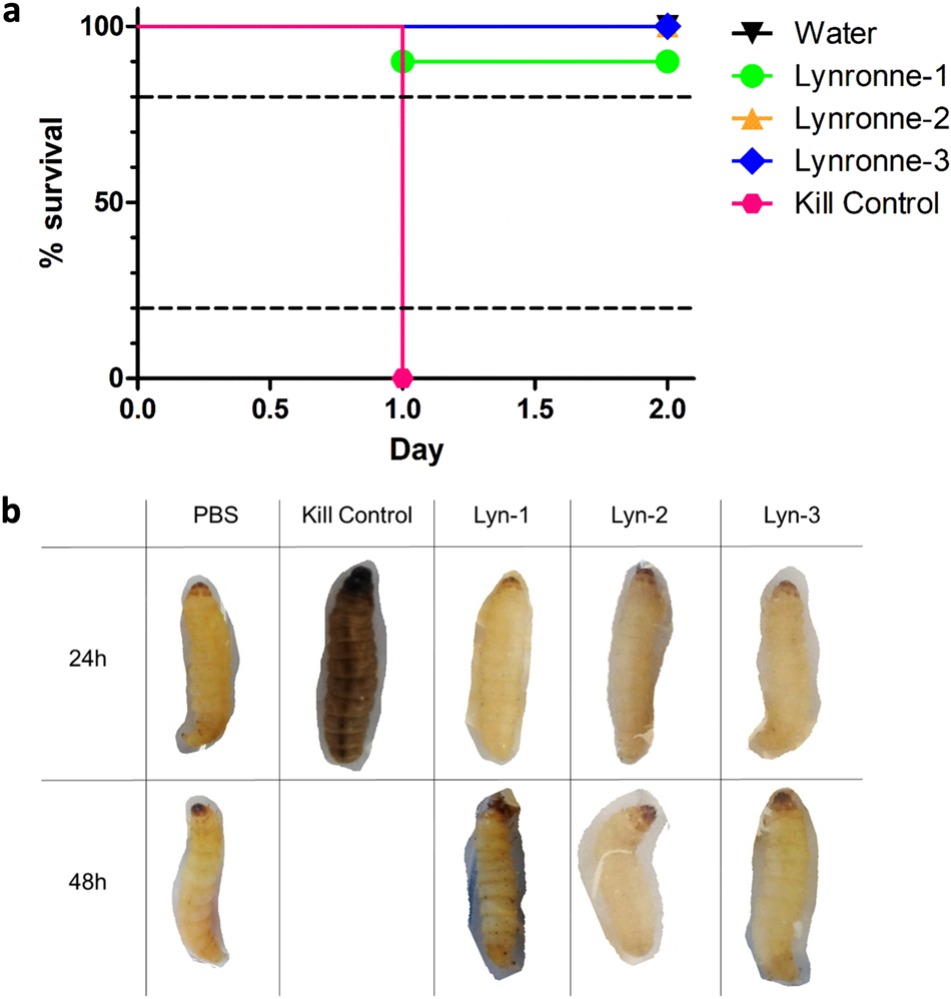
Toxicity determination of the Lynronne AMPs via *Galleria mellonella* injection model at 8x MIC. **a** Survival curves of the Lynronne AMPs across 48 h after injection. Kill controls were established with 10^7^ CFU/ml of *Acinetobacter baumannii* DSM 30007. **b** Table showing representative larval status at 24 h and 48 h after incubation at 37 °C. Status of larvae was determined using a combination of melanisation (as seen visibly in the 24 h Kill Control treatment), motility signs and responsiveness to physical stimuli. All treatments contained 10 replicates. >80% survival indicates low/negligible toxicity, between 20 and 80% indicates partial toxicity, and <20% survival indicates high toxicity.

The Lynronne peptides showed no toxicity in the waxmoth larvae (*Galleria mellonella*) up to the highest tested concentration of 8X MIC, with survival rates for Lynronne-1 at 90%, and Lynronne-2 and -3 demonstrating 100% survival over 48 h, which further validates their potential as treatment options for *A. baumannii* infections (Fig. 11a). Figure 11b shows representations of each treatment group after exposure.

## Discussion

In this study we tested the efficacy of antimicrobial peptides, Lynronne-1, Lynronne-2 and Lynronne-3 previously identified from a rumen microbiome^26^, for the first time against *A. baumannii* to establish whether these peptides would be viable candidates for the treatment of this critically important pathogenic bacterial species. We characterised their likely structural conformations, antimicrobial activity against planktonic and biofilm cells, synergistic potential, likelihood of resistance development and cytotoxicity, as well as elucidating their mode of action towards *A. baumannii*. We also investigated bacterial responses to AMP exposure using transcriptomics.

Structural examination using circular dichroism indicates that Lynronne-1, -2 and -3 all demonstrated alpha helical structures when in the presence of SDS detergent micelles, based on the observed peaks at 195 nm, and low dips at 208 nm and 220 nm^32^, suggesting that this conformation is also adopted during bacterial lipid binding corroborating with previous findings for Lynronne-1^28^. The difference in spectra peak height indicates that Lynronne-3 structures in SDS may contain less α-helical content than Lynronne-1 or -2. This may relate to the lower activity observed by Lynronne-3 against *A. baumannii*, with a less hydrophobic conformation being produced in contact with lipid membranes interfering with binding and disrupting.

MICs against a range of clinical *A. baumannii* isolates varied between 2 and 128 µg/ml, with >2 log_10_ reductions in CFU/mL within 30 min of exposure based on kill kinetics. MIC values were largely maintained for Lynronne-1 and -2 in the presence of elevated salt conditions; although increases were seen for Lynronne-3. Many AMPs of clinical interest (cathelidicins such as LL-37, AM-CATH28, WAM-1, naturally sourced peptides such as melittin and magainin-2) have been reported to inhibit *A. baumannii* growth at ranges between 4 and 64 μg/ml^33^ and therefore the Lynronne peptides (especially in the case of Lynronne-1 and Lynronne-2) are more effective, with MICs between 4 and 16 μg/ml against the tested strains.

Lynronne-2 and Lynronne-3 showed an ability to work additively with amoxicillin, erythromycin, gentamycin and tetracycline at 0.25× MIC, although no additive activity was observed when in combination with vancomycin. It was hoped that facilitating entry of the vancomycin molecule into the bacterial cell would allow for visible antimicrobial effects (not traditionally observed against *A. baumannii*). Given the size of vancomycin is approximately twice the size of any of the other tested antimicrobials (~1450 g/mol, erythromycin the second largest at ~730 g/mol), it could be hypothesised that any pore formation or membrane disruption caused by the Lynronne AMPs is not large enough to facilitate the entry of vancomycin into the cell.

To determine how well the Lynronne AMPs are at effecting the various survival mechanisms employed by *A. baumannii*, their performance against both biofilm formation, and previously established biofilms was examined. Lynronne -1, -2 and -3 showed significant effects against the formation and attachment of biofilms produced by three strains of *A. baumannii* (DSM 30007, DSM 102929 and S26063), with more limited (albeit significant) effects against previously established biofilms. In the context of more widely studied AMPs such as LL-37^34,35^, Lynronne-1 and -2 showed comparable performance against biofilms, though Lynronne-3 was able to significantly reduce the mass of established biofilms in comparison to LL-37. It should be noted that the crystal violet assay does not differentiate between live or dead cells and will stain all cellular material and extracellular matrix present in the well, so this does not provide data on metabolic status^30^. However, it is likely that the Lynronne peptides can reduce the numbers of viable cells present in the biofilm, given their previously suspected mode of action^26^. Previous studies have recommended the use of AMPs as ‘anti-biofilm peptides’, potentially in cleaning solutions or incorporated in the surfaces of plastics in clinical environments, as their rapid mode of action precludes the production of biofilm mass^36^. Therefore, Lynronne AMPs are strong candidates as anti-biofilm agents.

There was a slight rise in MIC concentrations during serial passage in resistance assays, although previously published research with the Lynronne peptides demonstrated limited resistance development when challenging MRSA or *P. aeruginosa*^25,26^. *A. baumannii* has been well established in adapting to hostile environments (notably with the production of persister cells and lowering of metabolic processes) which may explain the slightly greater ability to adapt when challenged with the AMPs. Nonetheless these increases in MIC would require further exploration to determine whether the rises in MIC were genotypic resistance development or short-term transient resistances.

Lynronne-1 and -3 act via membrane disruption against *A. baumannii* as demonstrated in the propidium iodide assay, with Lynronne-2 demonstrating membrane permeabilization effects only at supra-MIC concentrations^25,27^. This was complemented by the lipid interaction assays carried out using total lipid extracts from *A. baumannii* cells, in which Lynronne-1 and -3 both showed a higher affinity for lipid binding than Lynronne-2. This further confirms that Lynronne-1 and -3 are effective membrane disruptors, and that Lynronne-2 is capable of membrane disruption but is likely to employ alternative mechanisms of action. The results shown here also demonstrate that two of the three AMPs (Lynronne-2 and Lynronne-3) can also work in combination with some clinically significant antibiotics, notably gentamicin, likely due to the membrane disrupting mode of action that Lynronne-3 exhibits. Additionally, Lynronne-2 has been shown to have lower but significant lipid affinity in previous studies and may produce pores to facilitate gentamicin entry into the cell, thus highlighting the potential for these AMPs to be used in combination therapy for *A. baumannii* treatment. Many cationic AMPs work via binding and destabilisation of the bacterial membrane, but there are well-characterised AMPs with intracellular targets^37^, which fits with Lynronne-2 having a potential non-membrane targeting mechanism of action. This is hugely beneficial in regard to promoting the Lynronne AMPs as clinical candidates and can aid in the hindrance of antimicrobial resistance development^38^. The mechanism of action of Lynronne-2 will need to be further investigated for progression of this AMP for therapeutic use. Exploration into possible DNA/protein binding, or fluorescence-based imaging to determine AMP aggregation within the bacterial cell^39^ could help to shed light on the mechanisms deployed by Lynronne-2.

The transcriptomic analysis revealed that several genes were differentially expressed in *A. baumannii* cultures during AMP exposure. It was anticipated that genes linked to the adeABC/adeIJK efflux transporter family or *macA, macB* genes, previously linked to resistance responses to colistin, would be upregulated in response to peptide exposure^40^, but these genes were not differentially expressed in this study. Based on their adjusted *P* value (*P* < 0.01), the *csu* family (*csuA,B,C,D,E*, which form the chaperone-usher pili assembly system utilised for adherence in persister cell and biofilm formation^41^) were all upregulated after AMP exposure, with expression levels elevated more than 2-fold in all three AMP treatments. All three AMP treatments caused significant downregulation of trehalose-6-phosphate synthase encoded by the *OtsA* gene in *A. baumannii*, as identified by Iturriga et al. in 2009^42^. Trehalose is utilised by *A. baumannii* as a stress protector in the event of salt and heat stress, and trehalose-6-phosphate is the precursor to this sugar molecule^43^. Trehalose-6-phosphate, in high quantities, is believed to be toxic to the bacterial cell and has been linked to growth inhibition and a reduction in tolerance to elevated heat and/or salt conditions^44^. Reduction in this pathway may be an indication of the cell decreasing certain non-critical metabolic processes in response to rapid membrane damage. In comparison to the transcriptome changes caused by ciprofloxacin treatment, which targets DNA replication processes via the DNA gyrase protein, and which showed clear upregulation in genes linked to DNA damage and repair^45^, the Lynronne AMPs generally displayed downregulation or no change in the expression of similar genes. This indicates that should the Lynronne AMPs work intracellularly (as predicted in the case of Lynronne-2 in earlier studies^26^), it is unlikely that they have an impact on DNA replication.

Following the low haemolytic activity and negligible cytotoxicity against HaCaT, Caco-2, BEAS and A480 cell lines (representative of keratinocytes, epithelial cell morphologies and lung cells respectively, suitable screens for AMPs likely to be delivered via topical application, nebulised inhalation or orally), the toxicity of Lynronne-1, 2 and 3 were tested against the *G. mellonella* and observed to have no visible toxic effects within this complex system, with >90% survival at the highest tested concentration. This lack of toxicity demonstrated by Lynronne-1, -2 and -3 is a positive sign of their specificity towards bacterial cells, and their lack of activity towards eukaryotic cells and systems. Toxicity with linear AMPs has traditionally been a challenge, with some recent peptide advances being focused on modification of previously toxic AMPs, such as LL-37 and Magainin-II^46–48^. Whilst delivery of AMPs has usually been targeted towards topical and pseudo-topical treatments due to both their toxicity and poor bioavailability and stability^49,50^, the low toxicity against both BEAS (bronchial) and A480 (kidney) cell lines is a promising sign as further delivery mechanisms and treatment routes for *A. baumannii* infections could be employed such as oral and aerosol delivery for Lynronne AMPs.

These results in their entirety provide confirmation that rumen-derived AMPs, specifically, Lynronne-1, 2 and 3 from this provide a promising area of novel antimicrobial treatments for *A. baumannii*. Additionally, we show that gastrointestinal microbiomes, such as the rumen, are a valuable resource for the identification of future therapeutics targeting clinically relevant bacterial species. Developing novel antimicrobial treatments in the current AMR landscape is more critical than ever, and these therapeutics can provide a crucial resource for the improvement of future global health.

## Methods

### Antimicrobial agents

AMPs Lynronne-1, -2 and -3 were synthesised by GenScript (Netherlands) at 98% purity, and Ciprofloxacin (Sigma Aldrich, UK), used as the antibiotic control treatment, was dissolved in sterile water at required concentrations prior to use. Amoxicillin, erythromycin, gentamycin, tetracycline and vancomycin (all from Sigma Aldrich, UK), were used for determining synergistic MICs and were dissolved in sterile distilled water at required concentrations prior to use.

### Strains and growth conditions

Fourteen strains of *A. baumannii* were utilised for MIC/MBC testing (7 from the DSMZ culture collection; DSM 24110, DSM 30007, DSM 30008, DSM 30011 DSM 102929, DSM 102930, DSM 105126, and 7 clinical isolates obtained from Public Health Wales; S26063, S15785, S15908, S27379, S17658, S25722, S17910). Strains were streaked out from freezer stocks (in cation-adjusted Mueller Hinton broth (MH) containing 30% glycerol v/v (Sigma Aldrich, UK)) onto Mueller Hinton agar (Sigma Aldrich, UK) plates to obtain pure colonies. All assays were carried out in triplicate unless otherwise stated in cation-adjusted Mueller Hinton Broth. Unless otherwise stated, the type strain, *A. baumannii* DSM 30007, was utilised for standardisation and reproducibility.

### Structural analysis

The secondary structure of Lynronne-1, -2 and -3 was determined using far-UV CD^32^. Far-UV circular dichroism works via measuring the absorbance of right and left-handed polarised light between 180 and 250 nm, which demonstrates secondary structures such as alpha helices/beta-pleated sheets via positive/negative peaks^32^. This technique can also be used to distinguish tertiary structures when using near-UV wavelengths between 250 and 320 nm^51^. To determine the preferential solvent for determining structure as well as identifying whether there were any conformational changes based on the aqueous environment, two solvents- water and 30 mM SDS (BioRad, UK) were used during CD testing.

The AMPs were diluted into solution in sterile water or 30 mM SDS to a final concentration of 20 μg/ml, and 3 ml aliquoted into a quartz cuvette. Five measurements per sample were taken using a Jasco J815 Spectropolarimeter at intervals of 0.1 nm between 185 and 250 nm and averaged for the final spectrograph. Baselines of each solvent were taken and removed from the final readings. Raw millidegree (mdeg) readings were converted to mean elliptical residue before graph plotting in Graphpad Prism 5. Structures were determined based on previously published model spectra^52^.

### Minimum inhibitory concentration (MIC) determination

To investigate efficacy of the AMPs against the *A. baumannii* strains, a modified broth dilution method in sterile polypropylene 96 well microtiter plates was used to determine MICs, following the International Organisation for Standardisation 20776-1 standard for MIC determination^53^. Single bacterial colonies were grown overnight at 37 °C in cation-adjusted Mueller Hinton broth until an OD_600_ of >0.5 (previously determined to provide a CFU/ml of 10^7^–10^8^) was achieved, and then diluted to a 2× stock of the final CFU/mL of 5 × 10^5^ for use. Wells were prepared with 100 µl of sterile cation-adjusted Mueller Hinton broth (Sigma Aldrich, UK), with an additional 80 µl in the first wells of each row, for a final volume of 180 µl. Twenty microlitres of 10× final concentrations of AMP/antibiotic was added to the first well of each row. One hundred microlitres of AMP/MH broth from these wells were serially diluted. One hundred microlitres of the bacterial stock was then added to each well before incubation at 37 °C in a static incubator. The MIC value was defined as the lowest concentration of compound which inhibited visible growth of bacteria after 18–24 h. Each experiment was carried out in triplicate, and the median result taken as the MIC of each compound.

### Antimicrobial peptide efficacy in variable salt conditions

MICs were run as described above, in cation adjusted Mueller Hinton Broth with the addition of physiological concentrations of salts found in plasma; NaCl 150 mM, CaCl_2_ 2.5 mM, MgCl_2_ 1 mM, KCl 4.5 mM, NH_4_Cl 6 µM, ZnCl 8 µM, FeCl_3_ 4 µM (all salts from Sigma-Aldrich,UK). These were run individually, as well as in a combination containing all of the salts present, again in triplicate with median values taken as the MIC.

### Kill kinetics

To determine bacteriostatic or bactericidal activities of the AMPs, a time-kill assay was carried out against *A. baumannii* DSM 30007^54^. AMPs and ciprofloxacin were added to 1 ml of *A. baumannii* broth culture (in Mueller Hinton broth and OD adjusted to obtain ~1 × 10^7^ CFU/mL) at a final concentration of 4× MIC and incubated in an orbital shaker at 37 °C, 180 rpm. Samples at each time point were washed in 100 mM Tris-HCl (Sigma Aldrich, UK), serially diluted in 100 mM Tris-HCl and plated onto Mueller Hinton agar. Agar plates were incubated for up to 24 h, and CFU/mL calculated for each time point. This assay was carried out in triplicate. Each replicate was plated twice, and the CFU/ml calculated from the mean of each count.

### Synergistic effects of the AMPs

To identify whether there were observable synergistic interactions between the Lynronne AMPs and the clinical antibiotics amoxicillin, erythromycin, gentamycin, tetracycline and vancomycin, a checkerboard MIC assay^55^ was used with set concentrations of AMPs. Briefly, two antimicrobials are tested in double serial dilutions, and the concentration of each drug is tested both alone and in combination to determine the effect of the individual drug, as well as the effect produced by their combination^55^. The fractional inhibitory concentration Index (FIC) was used to identify synergy between compounds. This was calculated by using the original MIC divided by the synergistic MIC and an FIC Index was used to interpret effects, i.e., antagonistic effect when (FIC of >4), or additive when (FIC > 0.5 < 1), or synergistic effect when (FIC of ≤ 0.5), while indifference (FIC 1–4).

### Resistance selection

To determine whether resistance to Lynronne AMPs was likely to occur over time, serial passage of *A. baumannii* DSM 30007 in the presence of sub-MIC concentrations of the Lynronne AMPs was conducted over 28 days. The wells with the highest concentration of AMP containing visible bacterial growth were regrown to a CFU/ml of 10^5^ in sterile cation-adjusted MH broth and used as the stock culture for the next passage. The daily MIC result was also recorded.

### Activity against biofilm growth and adhesion

The ability of the AMPs to affect biofilm attachment, maturation and dispersion was measured using a 96 well biofilm model^56^. *A baumannii* DSM 30007, DSM 102929 and S26063 cultures were grown at 37 °C in cation-adjusted MH broth until a CFU/ml of 10^7^–10^8^ was achieved at which point cultures were diluted 1/100 in preparation for 96 well plate set up. To test the effects of the AMPs on biofilm attachment and maturation, sterile polystyrene 96 well plates were set up with 90 µl of culture dilution with 10 µl of AMP at a final concentration of 4× MIC concentrations, sealed and incubated in an incubating orbital shaker at 37 °C, 180 rpm for 48 h. The ability to disperse established biofilms was examined using a similar method. One hundred µl of bacterial culture (as described above) was transferred into sterile polystyrene 96 well plates, which were then sealed and incubated at 37 °C, 180 rpm for 48 h. Post incubation, wells were washed 3× with 150 µl sterile PBS to remove non-adherent cells, before 100 µl MH broth containing AMP (4× MIC) was added. Plates were further incubated for 24 h before staining. Biofilms were gently washed 3× in PBS to remove non-adherent cells, fixed with methanol, air dried and stained with 0.5% (w/v) crystal violet for 15 min whilst being shaken at 80 rpm. Dye was resolubilized with 33% (v/v) acetic acid. Plates were shaken at 80 rpm for 15 min to ensure even dye resolubilisation, and OD_570_ was measured to quantify biofilm adherence. 100% and 0% biofilm mass were established using the positive and negative controls. Statistical analysis was carried out using the GraphPad Prism 5 software, and differences between the positive control and treatments were identified using 1-way ANOVAs with Dunnetts post-test.

### Transcriptomic analysis

The effects of Lynronne AMPs on gene expression in *A. baumannii* was explored via transcriptomic analysis^25^. *A. baumannii* DSM 30007 cultures were grown in cation-adjusted MH Broth overnight at 37 °C to an OD_600nm_ of >0.5 before being diluted 1/50 into 20 ml fresh MH broth. These cultures were regrown to an OD_600nm_ of >0.25 to ensure sufficient cell mass. Cultures were then treated with 1× MIC of each peptide, alongside an untreated control, before being incubated for 1 h at 37 °C at 180 rpm. Once treated, 20 ml of a 1:1 ice cold ethanol/acetone mixture was added to each culture and thoroughly vortexed before being snap frozen in liquid nitrogen for storage at −80 °C. Cultures were defrosted on ice before centrifugation at 5000 × *g* for 10 min at 4 °C. The supernatant was removed, and the pellet was resuspended in 5 ml of 1% β-mercaptoethanol (Bio-Rad, USA) in order to denature any remaining RNAses. The resuspension was centrifuged at 10,000 × *g*, and the supernatant removed. To lyse the cells, cell pellets were resuspended in 200 µl TE buffer (1 mM EDTA, 10 mM Tris-HCl, 15 mg/ml lysozyme, pH 8.0) and 10 µl Proteinase K (Qiagen, UK) and incubated at room temperature in an orbital shaker for 10 min. Following incubation, cultures were centrifuged at 12,000 × *g* for 10 min, and 500 µl removed into a fresh tube per sample. RNA extractions were carried out using a Qiagen RNeasy Plus mini kit and protocol (Qiagen, UK). In short, for each sample, 700 µl of buffer RLT (containing 1% β-mercaptoethanol), and 700 µl of 70% ethanol were added to the supernatant, before 700 µl was removed and loaded onto a RNeasy spin column. The column was washed three times with the following buffers: 700 µl buffer RW1, 8000 × *g*, 15 s; 500 µl buffer RPE, 8000 × *g*, 15 s; 500 µl buffer RPE, 8000 x *g*, 120 s. RNA samples were eluted from the columns using 50 µl nuclease-free water, and samples were examined for purity (via 260 nm/280 nm and 260 nm/230 nm ratios of 1.8–2.2 and 1.7–2.3, respectively) and concentration using a NanoDrop One UC-vis spectrophotometer (ThermoFisher Scientific, USA). Samples were ribosomally depleted using the Ribo-Zero Plus rRNA depletion kit (Illumina, US), before being sequenced at >5 M paired end reads per sample using the Illumina Mi-Seq. Reads were deposited in the EMBI-EBL database under accession number PRJEB58102.

Raw reads were imported into the Geneious Prime software (Version 2022.2.2, https://www.geneious.com) and paired reads were trimmed and mapped to a publicly available annotated genome (*A. baumannii* ATCC 19606, accession number NZ_CP046654). Gene expression counts were calculated for each treatment replicate, before being compared against the untreated sample with DESeq2 using RStudio, as included in the previously mentioned Geneious Prime software version. The comparison data was exported into CSV format and genes expression changes identified via volcano plot (VolcaNoseR, Version 2.0, https://huygens.science.uva.nl/VolcaNoseR2). Genes highlighted as having 0 absolute confidence were removed at this stage, and the top 50 significant genes of interest were identified based on their adjusted P value for each individual treatment, and genes that were represented in more than one treatment had their results amalgamated to avoid overrepresentation. Additional genes highlighted in the literature as being linked to *A. baumannii* survival or resistance mechanisms were selected for inclusion. Heatmap figures displaying gene up-/down-regulation based on Log2 differential expression values were generated using the ClustVis (version 4.0, https://biit.cs.ut.ee/clustvis) online tool (Supplementary data, Fig. 1).

### Membrane permeability

Membrane permeabilisation effects of the Lynronne peptides were measured using 96 well plate fluorescence assays using propidium iodide^31^. Essentially, *A. baumannii* cultures were grown at 37 °C in cation-adjusted Mueller Hinton broth until a CFU/mL of 10^7^–10^8^ was achieved. Cultures were spun down at 4000 x *g* for 10 min, and pelleted cells resuspended in sterile PBS. Propidium iodide (Thermo-Fisher Scientific, US) was added to the resuspended cultures to a final volume of 30 mM and incubated at 37 °C for 15 min. Plates were prepared as in the MIC determination, and fluorescence readings by a CLARIOstar Plus (BMG Labtech, UK) with excitation/emission at 540 nm and 590 nm were taken every 5 min for 85 min. Positive and negative controls were achieved using cetyl-trimethyl-ammonium bromide (CTAB, 300 µM, Sigma Aldrich, UK) and sterile PBS. 100% permeabilisation was determined by the average CTAB fluorescence reading between 60 and 85 min.

### Transmission electron microscopy

Transmission electron microscopy was used to visualise any obvious changes in cell morphology when cells had been exposed to Lynronne AMPs. *A. baumannii* cultures were grown to a CFU/ml of 10^8^ cells and exposed to a 4× MIC concentration of each peptide for 60 min. Cells were pelleted at 5000 x *g* and washed 3× in PBS before an equal volume of primary fixative (2.5% glutaraldehyde, 0.1 M sodium cacodylate, pH 7.2, Sigma Aldrich, UK) was added. These were vortexed and stored at 4 °C.

Samples were then pelleted and washed using a 0.1 M sodium cacodylate wash buffer and resuspended in a secondary fixative (1% osmium tetroxide, 0.1 M sodium cacodylate, pH 7.2). The samples were then centrifuged and resuspended in 2% ultra-low gelling temperature agarose solution (Agar Scientific Ltd, UK). These samples were serially washed from 30 to 100% ethanol before transitioning into 100% LR White—Hard Grade resin (London Resin Company, UK), and polymerising at 60 °C. 60–80 nm thin sections were cut, and observed using a JEOL JEM1010 transmission electron microscope (JEOL Ltd, Japan) at 80 kV.

### Lipid insertion

The insertion of Lynronne-1, -2 -3 into bacterial lipids was quantified using reconstituted lipid monolayer as previously described^25–27^. Briefly, total lipids were extracted from overnight liquid cultures of *A. baumannii* (DSM 30007) using the Folch extraction procedure^57–59^. Using a 50 µl Hamilton syringe Total lipid extract was spread at the surface of PBS (pH 7.4, volume 800 µl) creating a lipid monolayer at the air–water interface. First, a dose-dependent assay was performed in which the initial surface pressure of the lipid monolayer was fixed at 30 ± 0.5 mN/m, corresponding to a lipid packing density theoretically equivalent to that of the outer leaflet of the cell membrane^56^. After 5–10 min of incubation allowing equilibration, increasing concentrations of peptides were injected into the PBS sub-phase using a 10 µl Hamilton syringe. The variation of the surface pressure (DeltaP) caused by peptide insertion was then continuously monitored using a fully automated microtensiometer (µTROUGH SX, Kibron Inc., Helsinki, Finland) until reaching equilibrium (usually within 15–25 min). In a second series of experiments, the critical pressure of insertion (Pc corresponding to the theoretical initial pressure at which no insertion can occurs) of each peptide was determined. The variation of pressure (DeltaP) caused by the injection of peptide (at 1 µg/ml final concentration) was measured at different values of the initial pressure (Pi) of lipid monolayer. Results were plotted as DeltaP as function of Pi and the critical pressure of insertion was graphically determined as the intercept of the linear slope with the X-axis when the DeltaP is equal to zero. All experiments were carried out in a controlled atmosphere at 20 ± 1 °C and data were analyzed using the Filmware 2.5 programme (Kibron Inc., Helsinki, Finland).

### Erythrocyte leakage

Haemolysis assays using red blood cells from whole human blood stored in K3-EDTA (Cambridge Bioscience, UK) were used to determine whether the AMPs showed any affinity for red blood cell membranes indicating cytotoxicity. The red blood cells were washed in sterile PBS before being aliquoted into 96 well plates. 10 µl of AMP at 10× chosen concentration was added to 90 µl of red blood cells per well. Gradients were established between 0.125 and 512 µg/ml for antimicrobial peptides. Plates were incubated for 1 h at 37 °C in a rotary incubator (100 rpm) before centrifugation. 90 µl of supernatant was transferred to a fresh 96 well plate and OD_450_ nm was measured. Positive controls were established using 0.1% Triton-X 100 (Sigma Aldrich, UK), which was determined using the same method with a gradient between 1 and 0.025%. This assay was carried out in quadruplicate. ED_50_ was calculated using GraphPad Prism 8 as the concentration of AMP that induced 50% haemolysis.

### Animal and human cells viability

The toxicity of the AMPs towards animal and human cells was evaluated using a resazurin assay as previously described^60,61^. Human cells used were A498 (ATCC® HTB-44 ^TM^), BEAS-2B (ECACC, Sigma Aldric), Caco-2 (ATCC® HTB-37 ^TM^), HaCaT (Creative Bioarray, USA), and HepG2 (ATCC® HB-8065 ^TM^). All cells were routinely cultured in Dulbecco’s modified essential medium (DMEM) supplemented with 10% foetal bovine serum (FBS), 1% L-glutamine and 1% antibiotics (Thermo Fisher Scientific, France). Cells were maintained in 25 cm^2^ flasks in a 5% CO_2_ incubator at 37 °C.

For carrying out the toxicity assay, cells were detached with trypsin–EDTA solution (Thermo Fisher Scientific, Fra). Cell density was measured using Malassez counting chamber and cells were finally seeded into 96-well cell culture plates (Greiner bio-one, Fra) at approximately 10,000 cells per well. After confluency was reached (2–3 days), media from wells was then changed and cells were exposed to increasing concentrations of AMPs diluted in culture media (from 0 to 1 mg/ml, 1:2 dilution), before 48 h incubation at 37 °C in a 5% CO2 incubator. Cell viability was evaluated using a resazurin-based in vitro toxicity assay kit (Sigma-Aldrich, Fra) following manufacturer’s instructions. Briefly, wells were emptied, and cells were treated with 100 µL of resazurin, diluted 1:10 in sterile PBS containing calcium and magnesium (PBS++, pH 7.4).

After incubation for 2 h at 37 °C, fluorescence intensity (excitation wavelength of 530 nm/emission wavelength of 590 nm) was measured using a Biotek microplate reader (Biotek, Synergy Mx, Fra). The fluorescence values were normalised by the negative control corresponding to untreated cells and were expressed as percent viability. The CC_50_ values of the peptides on cell viability (i.e. the concentration of peptides causing a reduction of 50% of the cell viability) were calculated using GraphPad® Prism 7 software (Graphpad, USA).

### In vivo AMP toxicity to *Galleria mellonella*

The waxmoth larvae (*Galleria mellonella*) was used to establish toxicity in the presence of an innate immune system^61^. The waxmoth larvae model is increasingly being used as a cheap and efficient in vivo model for testing novel antimicrobials, often as a precursor or alternative to murine models. All experiments used 10 larvae weighing between 250 and 350 mg with no signs of melanisation or previous injury/infection. To establish toxicity, 20 µl of AMP (suspended in water, at 8× MIC) was injected into the lower left proleg. The larvae were incubated at 37 °C and observed over 48 h. Larvae survival was determined on motility and response to stimuli. Vehicle controls of water and PBS were also utilised to monitor for physical trauma.

Larvae were monitored at 24–48 h to determine survival. Observations were taken every 60 min between 0 h and 6 h, then time points 24 h and 48 h were observed if necessary. Observed characteristics included darkening/melanisation, black spots to indicate infection, lethargy upon stimuli and an ability to correctly orient themselves after being overturned. Galleria were considered deceased if a lack of response to stimuli as well as full body melanisation were observed. Cut-off points of 80% and 20% survival were used to differentiate between full/partial/low survivability. Survival graphs were plotted in GraphPad Prism 5.

## Supplementary information

Supplementary data figure 1

## Data Availability

Sequencing reads were deposited in the EMBI-EBL database under accession number PRJEB58102 and all raw data are provided in a supplementary excel file. This allows access to all data arising from this study.
