# Supplementary material for: Microbiome-derived antimicrobial peptides show therapeutic activity against the critically important priority pathogen, Acinetobacter baumannii
Source: NPJ Biofilms Microbiomes. 2024 Sep 30;10:92. doi: 10.1038/s41522-024-00560-2 (PMC11443000; doi:10.1038/s41522-024-00560-2)
Supplement: Supplementary file 1 — Supplementary data figure 1 [file 41522_2024_560_MOESM1_ESM.pdf]

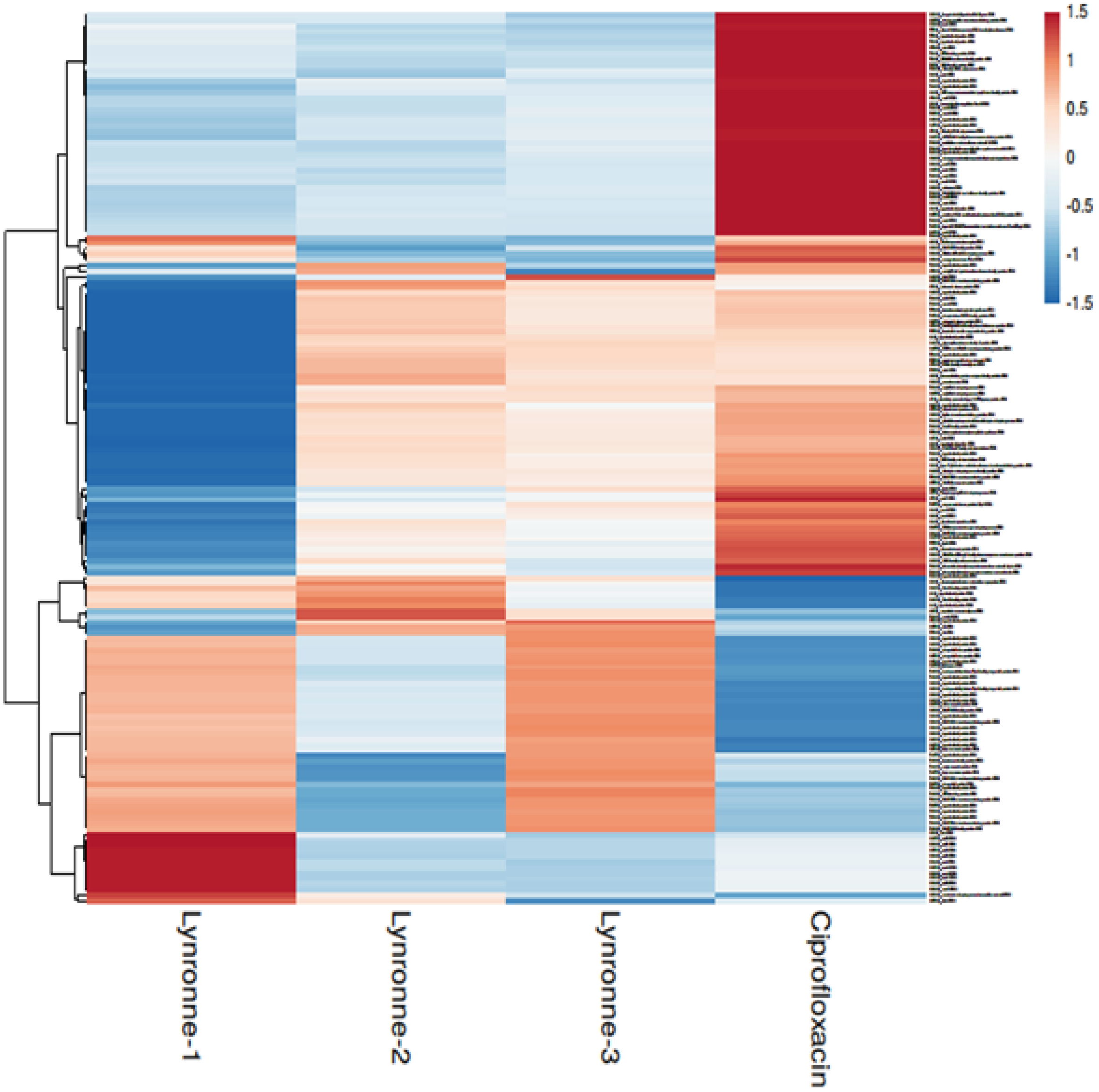


**Supplementary figure 1. Transcriptomic expression change comparison between Lynronne-1, Lynronne-2 and Lynronne-3 alongside ciprofloxacin.** The top 50 most changed genes as compared to an untreated control have been compiled for each treatment, and the heatmap generated using ClustalVis online webtool (Version 2.0, accessed on 10/11/2022 at https://biit.cs.ut.ee/clustvis/).
